# Extracellular vesicles from *Aggregatibacter actinomycetemcomitans* exhibit potential antitumorigenic effects in oral cancer: a comparative in vitro study

**DOI:** 10.1007/s00203-024-03976-8

**Published:** 2024-05-03

**Authors:** Marjut Metsäniitty, Shrabon Hasnat, Carina Öhman, Tuula Salo, Kari K. Eklund, Jan Oscarsson, Abdelhakim Salem

**Affiliations:** 1 Department of Oral and Maxillofacial Diseases, Clinicum, Faculty of Medicine, University of Helsinki, Helsinki, 00014 Finland; 2 Oral Microbiology, Department of Odontology, Umeå University, Umeå, 90187 Sweden; 3 Department of Rheumatology, University of Helsinki and Helsinki University Hospital, Helsinki, 00014 Finland; 4 Translational Immunology Research Program (TRIMM), Research Program Unit (RPU), Faculty of Medicine, University of Helsinki, Helsinki, 00014 Finland

**Keywords:** *Aggregatibacter actinomycetemcomitans*, *Porphyromonas gingivalis*, *Fusobacterium nucleatum*, *Parvimonas micra*, Extracellular vesicles, Oral cancer

## Abstract

*Aggregatibacter actinomycetemcomitans* is an opportunistic Gram-negative periodontopathogen strongly associated with periodontitis and infective endocarditis. Recent evidence suggests that periodontopathogens can influence the initiation and progression of oral squamous cell carcinoma (OSCC). Herein we aimed to investigate the effect of *A. actinomycetemcomitans*-derived extracellular vesicles (EVs) on OSCC cell behavior compared with EVs from periodontopathogens known to associate with carcinogenesis. EVs were isolated from: *A. actinomycetemcomitans* and its mutant strains lacking the cytolethal distending toxin (CDT) or lipopolysaccharide (LPS) O-antigen; *Porphyromonas gingivalis*; *Fusobacterium nucleatum*; and *Parvimonas micra*. The effect of EVs on primary and metastatic OSCC cells was assessed using cell proliferation, apoptosis, migration, invasion, and tubulogenesis assays. *A. actinomycetemcomitans*-derived EVs reduced the metastatic cancer cell proliferation, invasion, tubulogenesis, and increased apoptosis, mostly in CDT- and LPS O-antigen-dependent manner. EVs from *F. nucleatum* impaired the metastatic cancer cell proliferation and induced the apoptosis rates in all OSCC cell lines. EVs enhanced cancer cell migration regardless of bacterial species. In sum, this is the first study demonstrating the influence of *A. actinomycetemcomitans-*derived EVs on oral cancer in comparison with other periodontopathogens. Our findings revealed a potential antitumorigenic effect of these EVs on metastatic OSCC cells, which warrants further in vivo investigations.

**Supplementary Information:**

The online version contains supplementary material available at 10.1007/s00203-024-03976-8.

## Background

Oral squamous cell carcinoma (OSCC) is the most common malignancy of the oral cavity (Montero and Patel 2015). In 2020 alone, there were nearly 380,000 new cases and 180,000 deaths from oral cancer globally (Sung et al. 2021). Despite the progress in cancer diagnosis and management, the 5-year survival rate of OSCC remains relatively dismal without significant improvements over the past years (Economopoulou et al. 2019). Therefore, new therapeutic approaches are urgently needed to improve the survival outcomes of patients with OSCC.

Up to 20% of all human cancers are associated with microbial organisms, which can induce tumor-promoting chronic inflammation (Elinav et al. 2013). Recent accumulating evidence suggests that periodontopathogens may contribute to the initiation and progression of cancer (Metsäniitty et al. 2021; Teles et al. 2020; Xiao et al. 2020). The Gram-negative species, *Aggregatibacter actinomycetemcomitans* is associated with periodontitis and infective endocarditis (Nørskov-Lauritsen 2014). *A. actinomycetemcomitans* belongs to the HACEK group of bacteria which is a rare cause of infective endocarditis, responsible for 1–3% of all infective endocarditis. About 20% of the HACEK-induced endocarditis is caused by *A. actinomycetemcomitans* (Revest et al. 2016). The role of *A. actinomycetemcomitans* in cancer remains elusive. A recent meta-analysis reported that infection with *A. actinomycetemcomitans* as a single pathogen was not associated with increased risk of cancer (Xiao et al. 2020). On the contrary, *A. actinomycetemcomitans* showed a strong association with malignancy (Söder et al. 2021) and its virulence factors such as cytolethal distending toxin (CDT) and lipopolysaccharide (LPS) promoted pancreatic cancer (Ungureanu et al. 2023). In OSCC, CDT has been found to mediate anti tumor effects such as growth inhibition (Iwanaga et al. 2007), induction of apoptosis and cell cycle arrest (Yamamoto et al. 2004).

*A. actinomycetemcomitans* actively releases extracellular vesicles (EVs), also referred to as outer membrane vesicles, containing multiple virulence factors including for example the CDT (Faïs et al. 2016; Oscarsson et al. 2019; Rompikuntal et al. 2012), leukotoxin A (LtxA) (Kato et al. 2002; Kieselbach et al. 2015), outer membrane protein A, outer membrane protein 100, GroEL and peptidoglycan-associated protein (Kieselbach et al. 2015). Of interest, *A. actinomycetemcomitans* is—to our knowledge—the only known oral bacterial species producing the genotoxin CDT (Belibasakis et al. 2019), which has been implicated in the tumorigenesis of head and neck cancers (Damek-Poprawa et al. 2011; Iwanaga et al. 2007; Teshima et al. 2018; Yamamoto et al. 2004).

Lipopolysaccharide (LPS) is an abundant component of *A. actinomycetemcomitans*-derived EVs with immunomodulatory properties, hence representing an attractive target in cancer therapy (Jain et al. 2019; Shetab Boushehri and Lamprecht 2018; Song et al. 2018). The serotype-specific polysaccharide determinant of *A. actinomycetemcomitans* resides in the immunodominant LPS O-antigen, which differentiates the distinct serotypes of this species based on its antigenicity (Lakio et al. 2003; Oscarsson et al. 2019; Page et al. 1991; Sims et al. 1991; Wilson and Schifferle 1991). Lack of LPS O-antigen has been shown to alter both the pathogenic and immunostimulatory traits of *A. actinomycetemcomitans* (Lindholm et al. 2020; Monasterio et al. 2020). Importantly, LPS and CDT can be delivered into the host cells via EVs (Oscarsson et al. 2019; Rompikuntal et al. 2012; Vanaja et al. 2016).

Bacterial EVs are spherical bilayered proteolipids harboring multiple virulence and immunomodulator factors, which can be fully incorporated into the host cell cytoplasm (Kim et al. 2015; Ñahui Palomino et al. 2021). Therefore, EVs represent a promising target not only as drug delivery vehicles and bacterial vaccines but also in cancer therapeutics (Fazal and Lee 2021; Li et al. 2020). To our knowledge, only two studies explored the effect of bacterial EVs on oral cancer to date (Chen et al. 2023; Liu et al. 2021). In addition to *A. actinomycetemcomitans*, several EV-producing periodontopathogens have been linked to head and neck carcinogenesis such as *Porphyromonas gingivalis*, *Fusobacterium nucleatum* and *Parvimonas micra*. For instance, EVs from *P. gingivalis* showed pro-carcinogenic effects on metastatic OSCC cells (Liu et al. 2021). Furthermore, *P. gingivalis* influenced several tumorigenic events in OSCC including the epithelial-mesenchymal transition (EMT), tumor cell proliferation, invasion, metastasis, and angiogenesis (Lafuente Ibanez de Mendoza et al., 2020; Singh and Singh 2022). While *F. nucleatum* was associated with better outcomes in OSCC patients (Chen et al. 2020; Neuzillet et al. 2021), P. *micra* level was progressively increased from OSCC stage 1 to 4, implying a promising prognostic utility (Yang et al. 2018). Research on the role of *P. micra* in oral cancer is perhaps the most limited of the four periodontopathogens included in this study. In addition to the possible use of *P. micra* as a prognostic tool (Yang et al. 2018), *Parvimonas* (W.-H. Lee et al. 2017b; Zhao et al. 2017) and *P. micra* (Yang et al. 2018) abundance in saliva samples were associated with oral cancer and could differentiate patients with cancer from oral potentially malignant disorders (W.-H. Lee et al. 2017b). Thus bacterial species and their roles in carcinogenesis appear to vary among different individuals (Mager 2006).

Although recent evidence suggests a convincing link between oral dysbiosis and OSCC, the role of bacterial EVs in such association remains, however, unclear. Thus, we investigated whether and how EVs from different *A. actinomycetemcomitans* strains can influence the behavior of OSCC cells with variable metastatic potentials, compared to EVs from *P. gingivalis, F. nucleatum* and *P. micra.*

## Materials and methods

### Bacterial strains and growth conditions

Four strains of *A. actinomycetemcomitans* (serotype a) were used: D7SS is a natural genetic competent, smooth-colony derivative of wild-type strain D7S, which was originally isolated from a patient with aggressive periodontitis (Wang et al. 2002); and its *cdtABC* mutant derivative generated via a knockout approach (Nalbant et al. 2003) (hereafter referred to as D7SS-WT and D7SS-*cdt*, respectively). The strains SA3138 (Asikainen et al. 1995) and SA3139 (Asikainen et al. 1995; Kanasi et al. 2010) were isolated from a patient with periodontitis, albeit the latter lacks expression of the LPS O-antigen (hereafter referred to as SA3138-WT and SA3139-LPS-O, respectively).

*P. micra* CCUG 35243 and *F. nucleatum* CCUG 32989 were purchased from the Culture Collection University of Gothenburg; and *P. gingivalis* ATCC 33277 from the American Type Culture Collection. *A. actinomycetemcomitans* strains were cultivated in air supplemented with 5% CO_2_, at 37 °C on blood agar plates (5% defibrinated horse blood, 5 mg hemin/l, 10 mg Vitamin K/l, Columbia agar base; Oxoid Ltd., Basingstoke, Hampshire, UK) for 4 (D7SS strains) or 5 days (SA3138 and SA3139). *A. actinomycetemcomitans* strains can be cultured in trypsin soy broth, however, the EV protein pattern is very similar to the one of EVs from agar culture (Rompikuntal et al. 2012). *P. micra*, *F. nucleatum* and *P. gingivalis* were cultured in an anaerobic environment (10% H_2_, 5% CO_2_, 85% N_2_) at 37 °C. *P. micra* was cultured on blood agar plates for 5 days. *F. nucleatum* and *P. gingivalis* were first cultivated on blood agar plates for 2 and 3 days, respectively, and then the culture was continued in liquid broth fastidious anaerobe agar (FAA; Neogen®, Heywood, UK) for 48 h. All procedures were conducted in accordance with the guidelines of the local ethics committee at the Medical Faculty of Umeå University.

### Isolation of EVs

The EVs were isolated by ultracentrifugation as described earlier (Lindholm et al. 2020; Rompikuntal et al. 2012). In brief, bacterial cells were harvested from agar plates and suspended in 2 × 25 ml of phosphate-buffered saline (PBS) or liquid broth. The optical density (OD) values of the 25 ml suspensions at 600 nm were: 0.76 (D7SS-WT), 0.56 (D7SS-*cdt*), 1.12 (SA3138-WT), 1.38 (SA3139-LPS-O), 1.00 (*P. gingivalis)*, 1.36 (*F. nucleatum*) and 2.96 (*P. micra)*. The number of agar plates used for harvesting the bacterial cells was 5 (D7SS-*cdt)*, 10 (D7SS-WT, SA3138-WT and SA3139-LPS-O), and 30 (*P. micra*). The suspensions were centrifuged at 12,096 × *g* for 30 min at 4 °C in a JA-25.50 rotor (Beckman Instruments Inc.). Supernatants were filtered through a 0.45 and 0.2 μm pore-size syringe filters (Filtropur, Sarstedt) and centrifuged at 85,000 × *g* for 2 h at 4 °C in a 70 Ti rotor (Beckman Instruments Inc.). Pellets were washed twice with PBS (85.000 × g for 2 h at 4 °C) using a Sw60 Ti rotor (Beckman Instruments Inc.), resuspended in PBS, and used as EV preparation without further purification. EVs were tested for the absence of contamination by cultivating small aliquots on blood agar plates in air supplemented with 5% CO_2_ at 37 °C for 3 days.

### Analyses of EV preparation

EV protein concentration was determined using NanoDrop 100 spectrophotometer (Thermo Fisher Scientific) and the preparations were further analyzed using nanoparticle tracking analysis software Zetaview (Particle Metrix, Germany). To visualize proteins in EV samples, we performed a protein gel electrophoresis using Pierce™ Silver Stain Kit (Thermo Fisher Scientific) according to the manufacturer’s instructions. Samples were separated on Criterion™ TGX™ Precast Gels and Precision Plus Protein™ Standard All Blue (Bio-Rad) was used as a standard. Images were taken with ChemiDoc™ MP imaging system.

### Cancer cell lines and EV treatments

Two oral tongue cancer cell lines were used including primary SCC-24A (Department of Otorhinolaryngology, Head and Neck Surgery, Turku University Hospital, Finland) and highly metastatic HSC-3 cells (JCRB Cell Bank, Japan). Cell lines were cultured in 1:1 DMEM/F-12 medium supplemented with 10% heat-inactivated fetal bovine serum (Gibco), penicillin–streptomycin (Gibco), 250 ng/mL amphotericin B (Sigma-Aldrich, St. Louis, MO, USA), 50 µg/mL ascorbic acid (AppliChem, Chicago, IL, USA), and 0.4 µg/mL hydrocortisone (Sigma-Aldrich, St. Louis, MO, USA). For EV treatments, cells were challenged with EVs at the concentration of 5 µg/ml either once (6 h prior to the assay) or twice (6 h earlier and when initiating the assay; hereafter 2 × 5 µg/ml). The used EV concentration was based on recent literatures (Chen et al. 2023; Liu et al. 2021; Zhuang et al. 2023). Cells in the control wells were incubated in the same DMEM medium lacking EVs and referred to as no treatment controls (NTC). All incubations were done at 37 °C unless otherwise indicated.

### Real-time cancer cell proliferation and apoptosis assays

Cell proliferation and apoptosis assays were performed as previously described (Almahmoudi et al. 2019). OSCC cells were labelled with CellTrace™ Far Red dye according to the manufacturer’s instructions (Thermo Fisher Scientific) and then seeded in a 96-well plate (Corning) at a density of 2 × 10^3^ cells per well in 100 µl DMEM. The next day, media was replaced with fresh DMEM containing EVs (5 µg/ml) and incubated for 6 h. Then media was replaced with DMEM with or without EVs (5 µg/ml). Finally, the IncuCyte® Caspase-3/7 Green Apoptosis Assay Reagent (Cat. No. 4440) was added. The IncuCyte® Live-Cell Analysis System was used for imaging every 2 h for 2–3 days to assess cell proliferation and apoptosis.

### Transwell invasion assay

The effect of EVs on cancer cell invasion was assessed using Myogel-coated Transwell inserts (Corning Incorporated) as previously described (Salo et al. 2015). Briefly, inserts with 8.0 μm pore-size were coated with 50 µl Myogel (2.4 mg/ml) diluted in serum-free DMEM and solidified with rat tail type I collagen (0.8 mg/ml; Corning). Cells (70 × 10^3^/well) were seeded into the upper chambers in 200 µl serum-free DMEM supplemented with 0.5% lactalbumin and 5 µg/ml EVs. The lower chambers contained DMEM (500 µl) supplemented with 10% FBS. After 72 h, the invaded cells were fixed with 4% formaldehyde and stained with 1% toluidine blue in 1% borax. The dye was eluted with 1% SDS solution and absorbance was measured at 650 nm using the FLUOstar® Omega microplate reader (BMG Labtech). The invasion rate was calculated based on the measured absorbance.

### Cancer cell migration assay

Cell migration was assessed as recently reported (Karinen et al. 2021). First, a 96-well plate (Essen BioScience) was coated with 50 µl of Myogel (0.5 mg/ml) in serum-free DMEM and incubated overnight. Next, cells in 100 µl DMEM were plated at a density of 25 or 30 × 10^3^ per well (for HSC-3 and SCC-24A, respectively) and incubated overnight. Then DMEM was replaced with DMEM with or without EVs (5 µg/ml) and incubated for 6 h. Next, the WoundMaker™ (Essen BioScience) was used to obtain homogenous and consistent wounds. The wounds were washed 1–2 times with DMEM and after wound inspection DMEM with or without EVs (5 µg/ml) was added. The IncuCyte® Live-Cell Analysis system was used to image the wounds hourly until wound closure. The migration was analyzed using the relative wound density (RWD), which determines the density of the wound region relative to the density of the cell region, based on the initial scratch wound mask.

### Tube formation assay

Tube formation assay was performed as previously described (Francescone et al. 2011; Hujanen et al. 2021; Karinen et al. 2021). Briefly, 100 µl Matrigel® (8.9 mg/ml; Corning) was added to a 24-well cell culture plate (Corning) and incubated for 60 min at 37 °C to allow solidification. Cancer cells diluted in 300 µl serum-free DMEM were added on the gel-coated wells at a density of 10 × 10^4^ cells per well and incubated for 4 hours. Cells were then challenged with EVs (5 µg/ml) diluted in serum-free DMEM and incubation continued. Images were taken every 4, 8 and 20 h with ZEISS PrimoVert microscope (AxioCam ERc5s, Zeiss Microscopy) using magnifications 4x and 10x. The ImageJ software with “Angiogenesis Analyzer” plugin was used to measure the different tube formation parameters (Wayne Rasband, National Institute of Health, Bethesda, MD, USA).

### Statistical analyses

Statistical analysis was performed using GraphPad Prism Software version 9.4.1 (San Diego, California, USA). The One-way ANOVA with Dunnett`s or Tukey`s multiple comparisons test was used for proliferation, apoptosis, migration, and invasion assays. All experiments were repeated independently three times with triplicates or duplicates for each condition. Statistical significance was set to *P* ≤ 0.05; * indicates *P*-values ≤ 0.05; ** indicates *P*-values ≤ 0.01; *** indicates *P*-values ≤ 0.001; **** indicates *P* ≤ 0.0001. Data are represented as mean ± standard error of the mean (SEM).

## Results

### Characteristics of the EV samples

EVs used in this work were isolated from seven different bacterial strains as previously stated. Protein concentration of the EV samples varied between 0.931 and 9.391 mg/ml, while the particle diameter ranged from 132.96 to 161.16 nm (Table 1). Proteins were detected and visualized with Silver Stain protein gel electrophoresis where the protein sizes were compared to the pre-stained molecular weight marker (Fig. S1).

**Table 1:** Characteristics of the bacterial extracellular vesicles

Bacteria	Strain	Origin	Protein concentration* (mg/ml)	Particle concentration** (particles/ml)	Particle size** (diameter/nm)
*Aggregatibacter actinomycetemcomitans*	D7SS wild type	Patient	1.987	1.20 × 10^12^	143.52
*Aggregatibacter actinomycetemcomitans*	D7SS *cdtABC* mutant	Patient	1.258	5.2 × 10^11^	147.08
*Aggregatibacter actinomycetemcomitans*	SA3138 wild type	Patient	7.813	1.054 × 10^12^	139.22
*Aggregatibacter actinomycetemcomitans*	SA3139 lacking LPS O-antigen	Patient	8.732	1.6 × 10^12^	138.96
*Porphyromonas gingivalis*	ATCC 33277	ATCC	2.132	3.85 × 10^11^	133.16
*Fusobacterium nucleatum*	CCUG 32989 wild type	CCUG	0.931	3.66 × 10^10^	132.96
*Parvimonas micra*	CCUG 35243 wild type	CCUG	9.391	4.68 × 10^11^	161.16

*cdtABC*, cytolethal distending toxin subunit A, B and C gene; LPS, lipopolysaccharide; CCUG, Culture Collection University of Gothenburg; ATCC, American Type Culture Collection. *Protein concentration was measured with NanoDrop 100 spectrophotometer (Thermo Fisher Scientific). **Particle concentration and size were analyzed with nanoparticle tracking analysis software Zetaview (Particle Matrix, Germany)

### Effect of EVs from A. actinomycetemcomitans lacking CDT on cancer cell behavior

**Fig. 1:**
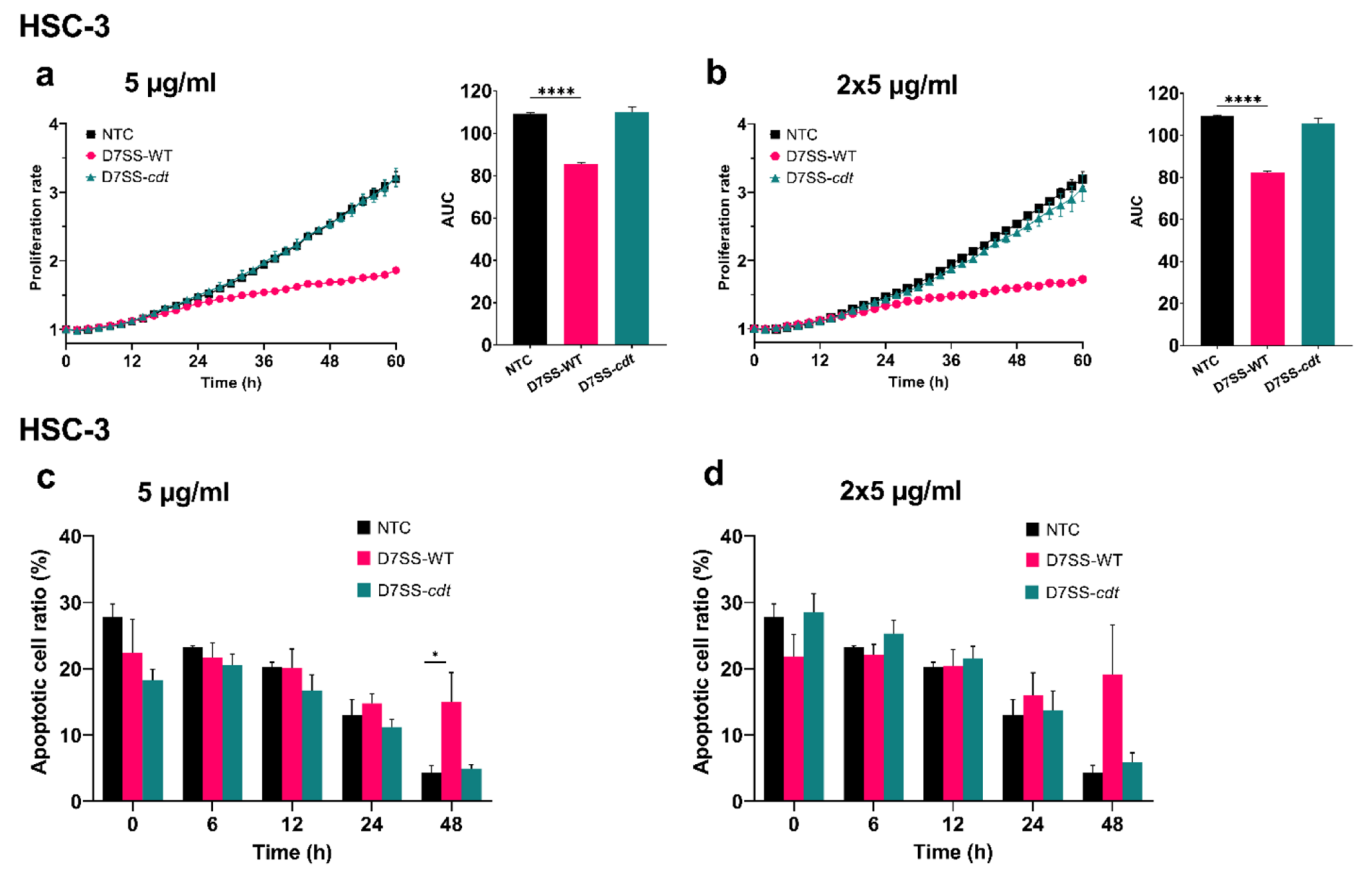
Effect of *A. actinomycetemcomitans* D7SS-WT and D7SS-*cdt* EVs on cancer cell proliferation and apoptosis. Cell proliferation rates are presented as proliferation rate in relation to time with the corresponding area under the curve (AUC). Apoptotic cell ratios are shown from representative time points. **(a, b)** HSC-3 cell proliferation was significantly inhibited by D7SS-WT EVs. (**c, d)** HSC-3 cell apoptosis levels were increased in cells treated with EVs from D7SS-WT compared to controls. Values are shown as mean ± SEM. ****P ≤ 0.0001. NTC, no treatment control. Experiments were repeated independently three times with triplicates for each condition

*A. actinomycetemcomitans* actively produces CDT, which has been shown to induce cell cycle arrest and apoptosis upon transfection into gingival squamous carcinoma cells (Belibasakis et al. 2019; Iwanaga et al. 2007). However, the tumorigenic effect of EVs from strains without this genotoxin is unknown. Thus, we first assessed the effect of EVs isolated from the wild-type *A. actinomycetemcomitans* (D7SS-WT) and its CDT-lacking derivative (D7SS-*cdt*) on cell proliferation and apoptosis of primary and metastatic OSCC cell lines. Interestingly, the D7SS-WT-derived EVs (at 5 µg/ml and 2 × 5 µg/ml) remarkably reduced the metastatic HSC-3 cell proliferation (*P* < 0.0001), while no effect was observed by the D7SS-*cdt*-derived EVs (Fig. 1a, b). Notably, an increased rate of apoptosis was observed in HSC-3 cells treated with the D7SS-WT-derived EVs (5 µg/ml) compared with the untreated controls (*P* < 0.05) at 48 h (Fig. 1c). Similarly, the apoptosis ratio was increased in HSC-3 cells at 48 h following treatment with the D7SS-WT-derived EVs (2 × 5 µg/ml), however, the difference was not statistically significant (Fig. 1d). Of note, none of the EVs significantly altered neither the proliferation nor the apoptosis levels of the primary SCC-24A cells (Fig. S2a-d).

**Fig. 2:**
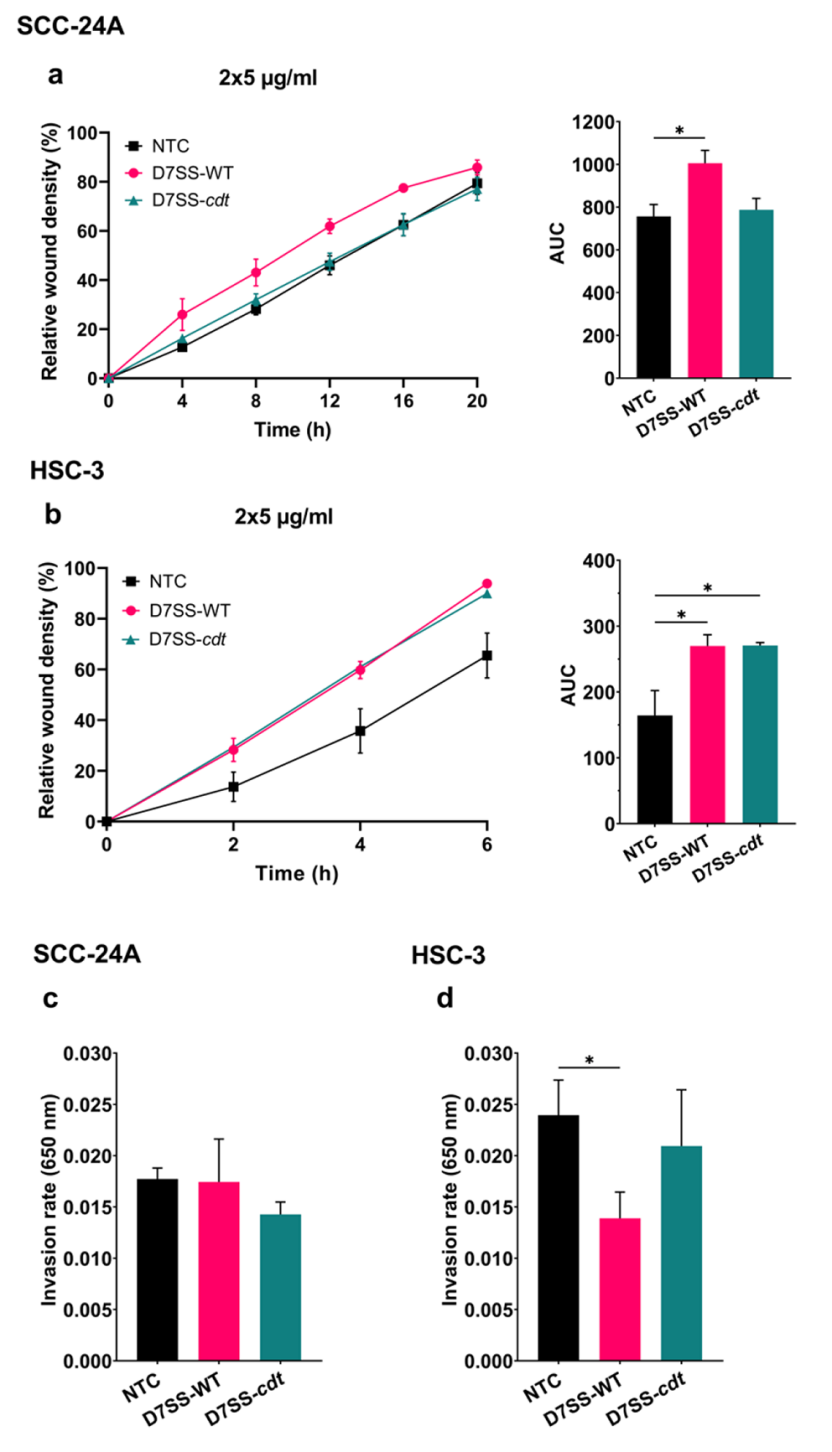
Effect of *A. actinomycetemcomitans* D7SS-WT and D7SS-*cdt* EVs on cancer cell migration and invasion. **(a, b)** Cell migration is presented as the relative wound density over time with the corresponding area under the curve (AUC). **(a)** SCC-24A cell migration was induced only by EVs (2 × 5 µg/ml) from D7SS-WT. **(b)** HSC-3 cell migration was increased when cells were treated with D7SS-WT and D7SS-*cdt* EVs. **(c, d)** Transwell invasion of SCC-24A and HSC-3 cells treated with EVs (5 µg/ml) from D7SS-WT and D7SS-*cdt*. **(c)** SCC-24A invasion was not significantly affected by EVs. **(d)** HSC-3 cell invasiveness was decreased by D7SS-WT EVs but not by D7SS-*cdt* EVs. Values are shown as mean ± SEM. *P ≤ 0.05. NTC, no treatment control. Experiments were repeated independently three times with triplicates (migration) or duplicates (invasion) for each condition

We then evaluated the impact of these EVs on cancer cell migration and invasion. To this end, we first quantified the RWD metric using the IncuCyte® Live-Cell Analysis, which revealed a modest increase in the SCC-24A cell migration upon treatment with the D7SS-WT EVs (2 × 5 µg/ml; *P* < 0.05) (Fig. 2a). The metastatic HSC-3 cell migration was enhanced by EVs (2 × 5 µg/ml) from both strains (*P* < 0.05) (Fig. 2b). However, EVs at the concentration of 5 µg/ml showed no statistically significant effect on cancer cell migration (Fig. S2e, f). The Myogel-coated Transwell chambers were then used to study the effect of bacterial EVs on OSCC cell invasion. Interestingly, none of the EVs affected the invasiveness of the primary cells (Fig. 2c), and only those obtained from the D7SS-WT strain significantly blunted the metastatic cell invasion (*P* < 0.05) (Fig. 2d).

### Effect of EVs from A. actinomycetemcomitans lacking LPS O-antigen on cancer cell behavior

**Fig. 3:**
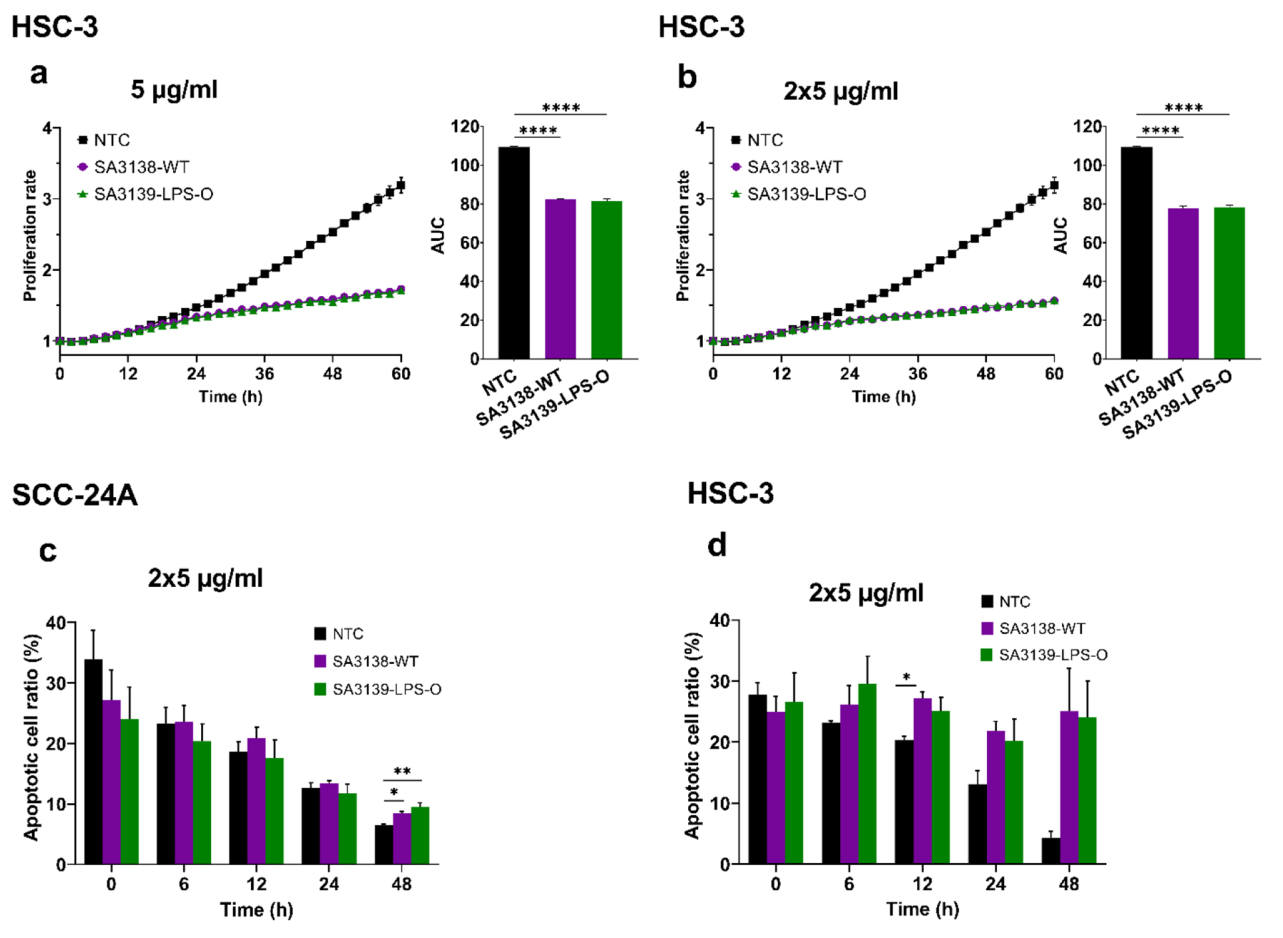
Effect of EVs from *A. actinomycetemcomitans* SA3138-WT and SA3139-LPS-O strains on cancer cell proliferation and apoptosis. Cell proliferation rates are presented as proliferation rate in relation to time with the corresponding area under the curve (AUC). Apoptotic cell ratios are shown from representative time points. **(a, b)** HSC-3 cell proliferation was significantly inhibited by SA3138-WT and SA3139-LPS-O-derived EVs (5 µg/ml and 2 × 5 µg/ml). **(c)** At 48 h the primary SCC-24A cells treated with SA3138-WT and SA3139-LPS-O-derived EVs (2 × 5 µg/ml) had significantly higher apoptosis rate than control cells. **(d)** The metastatic HSC-3 cell apoptosis was significantly increased in cells treated with EVs from SA3138-WT at 12 h of treatment. Values are shown as mean ± SEM. ****P ≤ 0.0001. *P ≤ 0.05. NTC, no treatment control. Experiments were repeated independently three times with triplicates for each condition

LPS O-antigen plays a key role in *A. actinomycetemcomitans* virulence (Monasterio et al. 2020). However, it is unknown whether it can also have an impact on cancer cell behavior. Therefore, we next challenged OSCC cells with EVs from the wild-type *A. actinomycetemcomitans* (SA3138-WT), and a strain lacking the O-antigen polysaccharide (SA3139-LPS-O), as described above. Our results showed that the proliferation of HSC-3 cells was significantly inhibited by EVs from both strains (*P* < 0.0001; Fig. 3a, b). On the contrary, none of these EVs significantly altered the proliferation of SCC-24A cells (Fig. S3a, b). For the programmed cell death, treatment with these EVs (2 × 5 µg/ml) showed a significantly higher apoptosis levels in SCC-24A cells at 48 h (SA3138-WT, *P* < 0.05; SA3139-LPS-O, *P* < 0.01; Fig. 3c), and in HSC-3 cells at 12 h (SA3138-WT, *P* < 0.05; Fig. 3d). In contrast, EVs (5 µg/ml) from SA3139-LPS-O reduced the apoptosis of SCC-24A cells at 24 h (*P* < 0.05), but no significant changes were observed in HSC-3 cells (Fig. S3c, d).

**Fig. 4:**
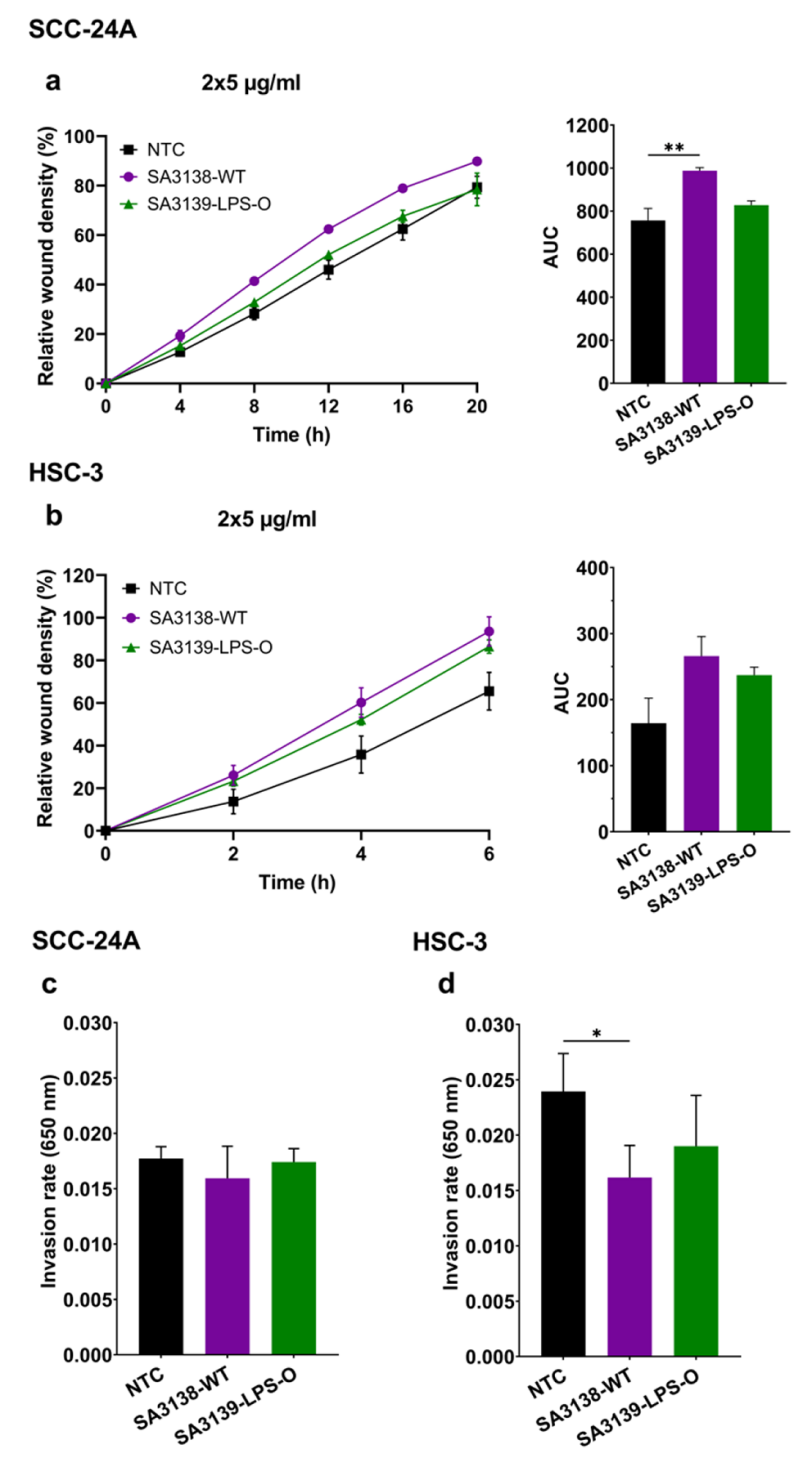
Effect of *A. actinomycetemcomitans* SA3138-WT and SA3139-LPS-O EVs on cancer cell migration and invasion. **(a, b)** Cell migration is presented as the relative wound density over time with the corresponding area under the curve (AUC).**(a)** SA3138-WT EVs increased SCC-24A cell migration compared to control. **(b)** HSC-3 cell migration was not significantly induced by EVs. **(c, d)** Transwell invasion of SCC-24A and HSC-3 cells treated with 5 µg/ml of SA3138-WT and SA3139-LPS-O EVs. **(c)** SCC-24A cell invasion was not affected by EV treatment. **(d)** SA3138-WT EVs reduced HSC-3 cell invasiveness. Values are shown as mean ± SEM. ** *P* ≤ 0.01. NTC, no treatment control. Experiments were repeated independently three times with triplicates (migration) or duplicates (invasion) for each condition

The migration of SCC-24A cells was significantly enhanced by the wild-type SA3138-WT-derived EVs (2 × 5 µg/ml; *P* < 0.05; Fig. 4a). EVs (2 × 5 µg/ml) from both strains appeared to promote HSC-3 cell migration, although the differences were not statistically significant (Fig. 4b). Likewise, no significant effect was observed on the migration of OSCC cell lines when treated with the 5 µg/ml EV-dose (Fig. S3e, f). Of interest, the invasion of the primary SCC-24A cells was not affected by EVs from either strain (Fig. 4c). In turn, the invasion of the metastatic HSC-3 cells was significantly reduced only by EVs from the SA3138-WT (*P* < 0.05) (Fig. 4d).

### Effect of A. actinomycetemcomitans-derived EVs on cancer cell tubulogenesis

**Fig. 5:**
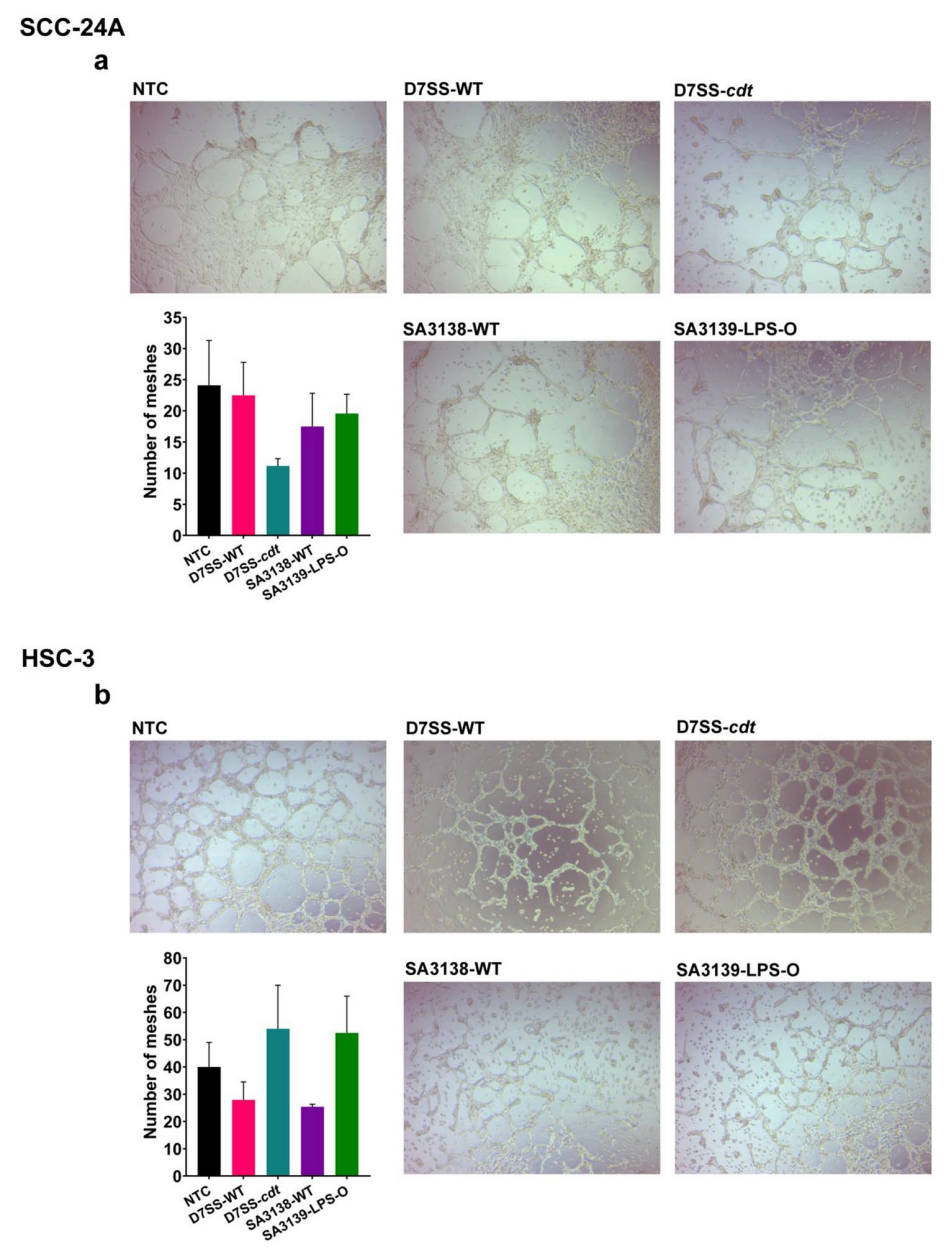
Cancer cell-derived tubulogenesis in SCC-24A and HSC-3 cells treated with *A. actinomycetemcomitans* EVs. **(a)** Tube formation in SCC-24A cells was partly inhibited by D7SS-*cdt*-derived EVs. **(b)** EVs from the wild-type strains (D7SS-WT and SA3138-WT) decreased tube formation in the metastatic HSC-3 cells. Values are shown as mean ± SEM. NTC, no treatment control. Experiments were repeated independently three times with duplicates for each condition

Recently, certain aggressive OSCC cells were shown to express the endothelial cell marker CD31 and initiate vascular networks similar to the endothelial cell tubulogenesis when cultured on biological hydrogels (Hujanen et al. 2021). These capillary networks were suggested as a possible mechanism behind metastasis and drug resistance in cancer patients (Williamson et al. 2016). Thus, we aimed to study the effect of EVs from *A. actinomycetemcomitans* on this process using the tube formation assay. After seeding on Matrigel® for four hours, cancer cells were incubated for two days with or without each of the four *A. actinomycetemcomitans*-derived EVs at 5 µg/ml. Overall, the primary SCC-24A cells formed fewer tubes on Matrigel® compared to the metastatic HSC-3 cells. Here we show representative images (4x) of tubulogenesis following 20 h of incubation with or without EVs (Fig. 5). All the tube-formation parameters are provided in the supplementary materials (Fig. S4-7).

EVs from the CDT-lacking strain (D7SS-*cdt*) resulted in less tube formation (i.e., number of meshes) by SCC-24A compared to those from the wild-type and untreated controls, whereas a little effect was observed with EVs from the SA3138-WT and SA3139-LPS-O strains (Fig. 5a). Interestingly, EVs from the wild-type strains (D7SS-WT and SA3138-WT) inhibited the tube formation by the metastatic HSC-3 cells compared to those from their mutant variants (D7SS-*cdt* or SA3139-LPS-O) and the untreated controls (Fig. 5b). Also, these results possibly suggest that CDT expression may exhibit an opposite effect of on the tubulogenic potential between primary and metastatic cancer cells. Although this finding should be interpreted with caution, it warrants further detailed investigation on the role of CDT.

### Effect of EVs from P. Gingivalis, F. Nucleatum and P. micra on cancer cell behavior

The periodontopathogens *P. gingivalis, F. nucleatum and P. micra* have been gaining attention for their association with multiple cancers including OSCC (Metsäniitty et al. 2021; Yang et al. 2018). However, the contribution of their EVs to the specific processes involved in oral carcinogenesis remain partly unknown. Thus, by employing the same OSCC in vitro model, we next aimed to explore how EVs from these bacteria can affect cancer cell behavior, compared to the findings from the *A. actinomycetemcomitans* strains.

**Fig. 6:**
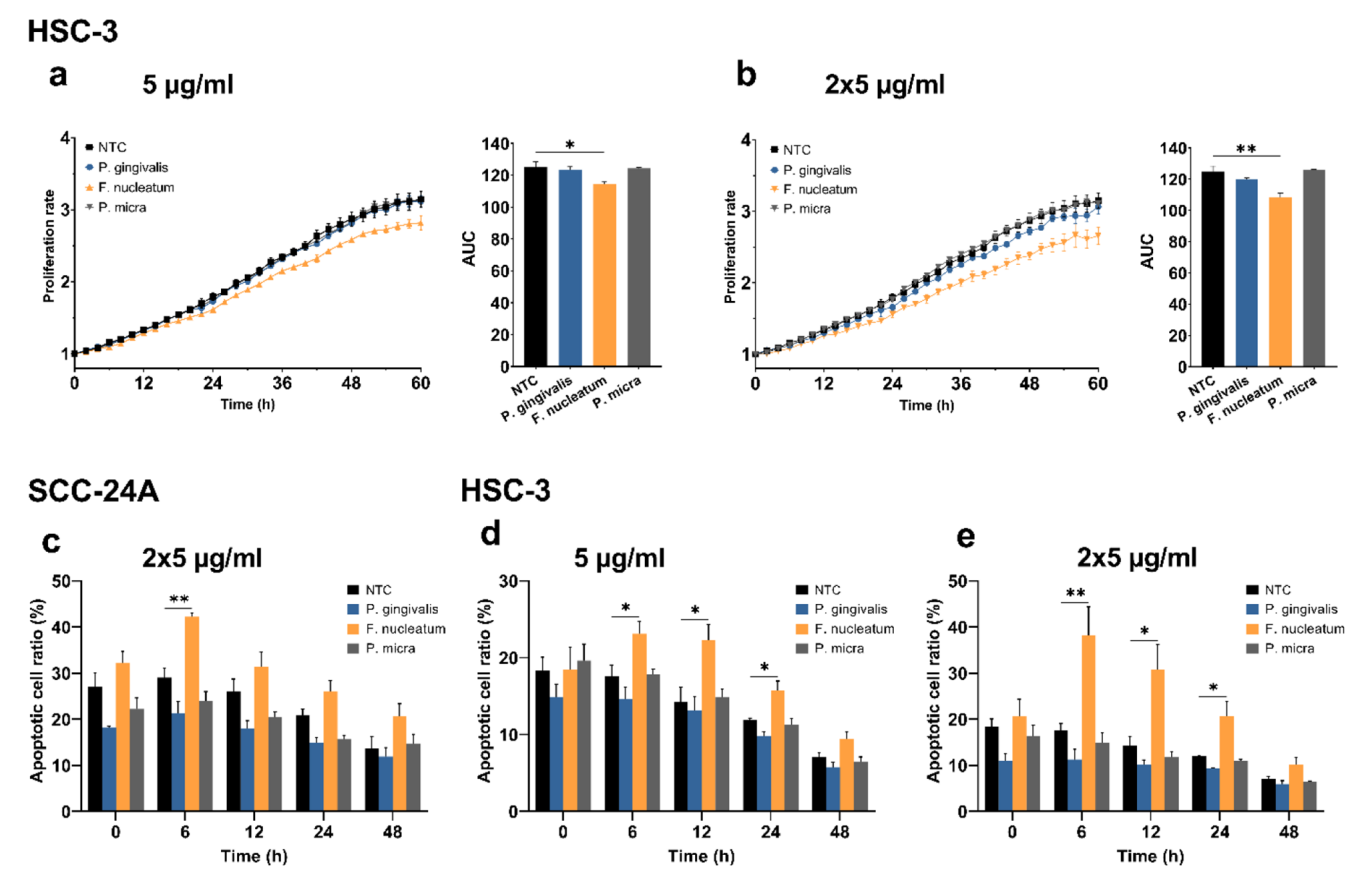
Effect of EVs from *P. gingivalis*, *F. nucleatum* and *P. micra* on cancer cell proliferation and apoptosis. Cell proliferation rates are presented as proliferation rate in relation to time with the corresponding area under the curve (AUC). Apoptotic cell ratios are shown from representative time points. **(a, b)***F. nucleatum*-derived EVs reduced HSC-3 cell proliferation at both doses. **(c)** SCC-24A cell apoptosis was significantly higher in cells treated with *F. nucleatum* EVs (2 × 5 µg/ml) at 6 h. **(d, e)** Both doses of *F. nucleatum* EVs significantly increased apoptotic cell ratio of HSC-3 cells at 6, 12, and 24 h. Values are shown as mean ± SEM. *P ≤ 0.05. **P ≤ 0.01. NTC, no treatment control. Experiments were repeated independently three times with triplicates for each condition

We report that only EVs from the *F. nucleatum* significantly inhibited the proliferation of HSC-3 cells (5 µg/ml, *P* < 0.05; 2 × 5 µg/ml, *P* < 0.01; Fig. 6a, b). None of the EVs affected the proliferation of SCC-24A cells (Fig. S8a, b). The *F. nucleatum*-derived EVs induced more cancer cell apoptosis, with the peak effect observed as follows: SCC-24A cells at 6 h (2 × 5 µg/ml EVs, *P* < 0.01; Fig. 6c); HSC-3 cells (5 µg/ml EVs at 6, 12 and 24 h, *P* < 0.05; Fig. 6d), and (2 × 5 µg/ml EVs at 6 h, *P* < 0.01; 12 and 24 h, *P* < 0.05; Fig. 6e). EVs from *P. gingivalis* showed a modest tendency to resist cell apoptosis although it was not statistically significant (Fig. 6c-e).

**Fig. 7:**
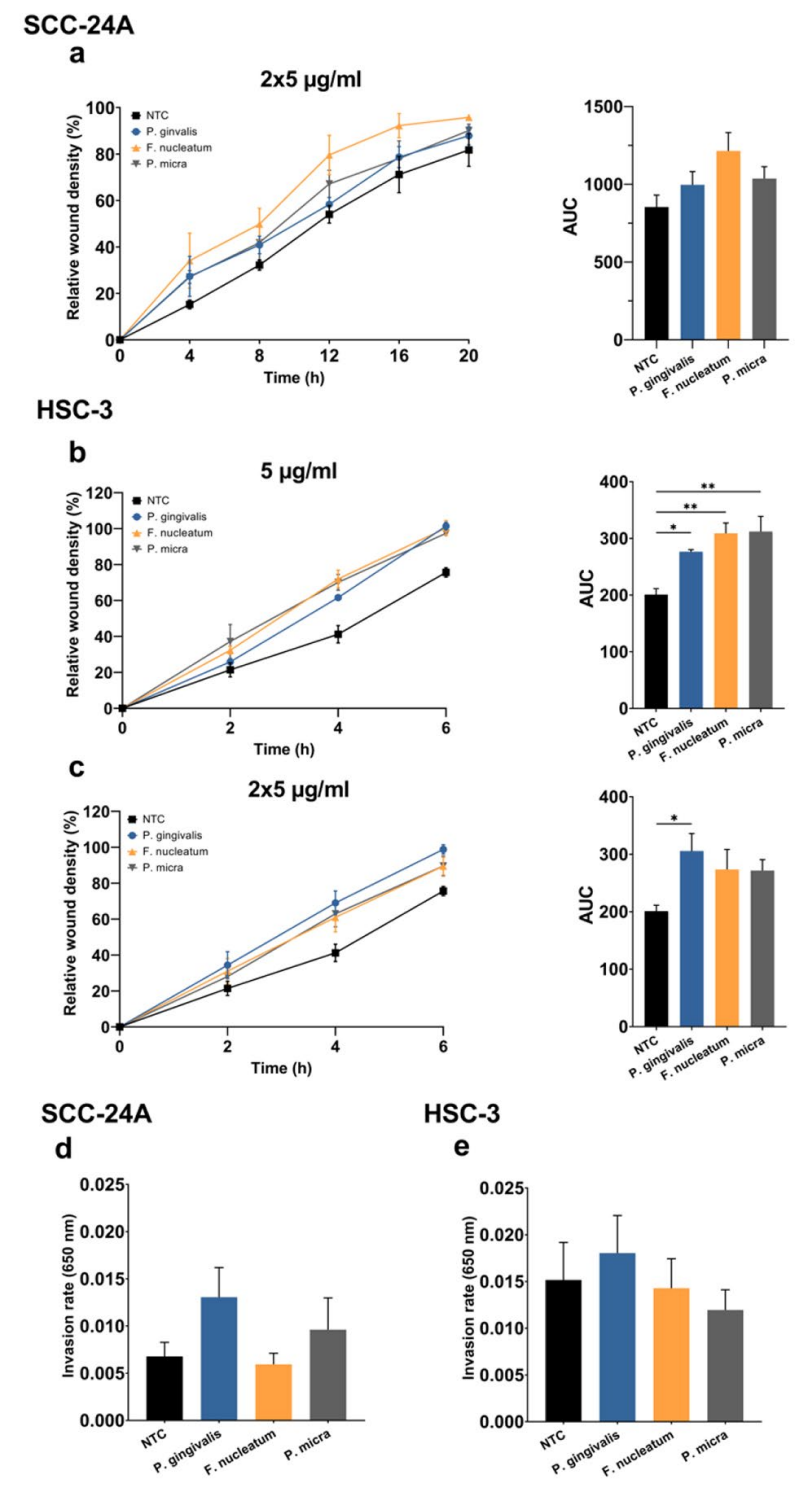
Effect of EVs from *P. gingivalis*, *F. nucleatum* and *P. micr*a-derived on cancer cell migration and invasion. **(a, b, c)** Cell migration is presented as the relative wound density over time with the corresponding area under the curve (AUC). **(a)***F. nucleatum* EVs (2 × 5 µg/ml) slightly promote SCC-24A cell migration, though the difference is not statistically significant (*P* = 0.055). **(b)***P. gingivalis*, *F. nucleatum* and *P. micra* EVs (5 µg/ml) increase HSC-3 cell migration. **(c)***P. gingivalis* EVs (2 × 5 µg/ml) significantly promoted HSC-3 migration. **(d, e)** Transwell invasion of SCC-24A and HSC-3 cells treated with 5 µg/ml EVs derived from *P. gingivalis*, *F. nucleatum* and *P. micra* did not show any statistically significant differences. Values are shown as mean ± SEM. *P ≤ 0.05. NTC, no treatment control. Experiments were repeated independently three times with triplicates (migration) or duplicates (invasion) for each condition

The IncuCyte® Live-Cell Analysis revealed a marginally significant increase in the migration of SCC-24A by EVs (2 × 5 µg/mg) from *F. nucleatum* compared to control cells (*P* = 0.055; Fig. 7a). HSC-3 cell migration was enhanced by EVs from *P. gingivalis* (both 5 and 2 × 5 µg/mg; *P* < 0.05), *F. nucleatum* and *P. micra* (5 µg/mg; *P* < 0.01) (Fig. 7b, c). Finally, we evaluated the effect of these pathogens on OSCC cell invasion using the Transwell assay. Although statistically significant differences were not observed, EVs from *P. gingivalis* and *P. micra* seemed to promote SCC-24A cell invasion (Fig. 7d), while *P. gingivalis* slightly enhanced the invasiveness of HSC-3 cells (Fig. 7e).

## Discussion

The present work is one of the first studies to investigate the interactions between bacterial EVs and OSCC cells in vitro. We showed that *A. actinomycetemcomitans*-derived EVs inhibited the proliferation and invasion of the highly metastatic HSC-3 cells and blunted their tubulogenic potential, mostly in CDT and LPS O-antigen dependent manners. Further, our analysis revealed that EVs from *F. nucleatum* suppressed the proliferation of OSCC cells and increased their apoptosis rate. All EVs tested in this work promoted the migration of cancer cells.

Previously, it was reported that incubation with *A. actinomycetemcomitans* caused up to 50% decrease in OSCC cell proliferation (Hoppe et al. 2016). Moreover, Teshima et al. showed that OSCC cell infection with *A. actinomycetemcomitans* induced CDT-dependent DNA double-strand breaks, which occurred independently of apoptosis (Teshima et al. 2018). Although such DNA breaks suggest a pro-carcinogenic activity, several studies have reported CDT-mediated antitumorigenic effects in oral cancer. For instance, transfection of a *cdtB*-expressing plasmid to OSCC cells enhanced cell cycle arrest and apoptosis in vitro and in vivo (Iwanaga et al. 2007; Yamamoto et al. 2004). Furthermore, a combination of *A. actinomycetemcomitans*-derived CDT with CD133 monoclonal antibody inhibited the proliferation of the aggressive CD133^+ ve^ oral cancer stem cells (Damek-Poprawa et al. 2011). Consistently, our findings revealed a potential anticancer effect of *A. actinomycetemcomitans*-derived EVs on metastatic OSCC cells in a CDT-dependent manner.

The activity of LPS on cancer cells is mostly mediated by host-dependent mechanisms (Lundin and Checkoway 2009). LPS activates toll-like receptors (TLRs) on cancer cells leading to a tumor-promoting environment or, alternatively, antitumor immune responses (Basith et al. 2012; Hasnat et al. 2020). LPS can also be delivered into the host cell cytosol via EVs causing pyroptosis and subsequent release of inflammatory cytokines (Vanaja et al. 2016). Here we showed that EVs from *A. actinomycetemcomitans* strains with and without LPS O-antigen impacted cancer cell behavior. These findings suggest that such structural change of LPS may, at least in part, influence oral tumorigenesis. Interestingly, cancer cell migration was the only process enhanced by all EVs, regardless of the bacterial species. The exact reason is not clear, however, a recent study showed that OSCC cell migration was enhanced by key periodontopathogens, including *P. gingivalis* and *F. nucleatum*, via integrin alpha V and FAK activation (Kamarajan et al. 2020).

Cancer cell-derived capillaries or vasculogenic mimicry (VM) have been linked with metastasis and poor survival of OSCC patients (Hujanen et al. 2020). In this context, it is interesting that *A. actinomycetemcomitans*-derived EVs attenuated the formation of these capillaries by HSC-3 cells, given their high tubulogenic potential across different matrices (Hujanen et al. 2021). Nevertheless, it remains to be elucidated why strains with and without CDT had an opposite effect on the tubulogenesis of primary and metastatic cancer cells, which warrants further mechanistic insights.

*A. actinomycetemcomitans* produces LtxA that is specific for human white blood cells by interacting with lymphocyte function antigen-1 on susceptible cells. Though LtxA was not included in this study it is noteworthy to mention that it might expose anti-cancer effects. For example, LtxA has been shown to kill malignant white blood cell lines and primary cells isolated from acute myeloid leukemia patients. Healthy peripheral blood mononuclear cells in turn were relatively resistant to LtxA (Kachlany et al. 2010). Even anti-lymphoma activity of LtxA was reported as it caused regression of B-cell tumors in mice (DiFranco et al. 2015). This demonstrates that *A. actinomycetemcomitans* has also other virulence factors aside from CDT and LPS that interact with cancer and possess therapeutic utility.

The effect of *F. nucleatum* on OSCC cell proliferation is conflicting, showing both stimulatory (Binder Gallimidi et al. 2015) and non-stimulatory effects (Kamarajan et al. 2020). Herein, we reported anticancer activities of *F. nucleatum* EVs by suppressing the proliferation and inducing the apoptosis of OSCC cells. Our findings support recent observations that higher tumoral levels of *F. nucleatum* were associated with better clinical outcomes in patients with head and neck cancers (Chen et al. 2020; Neuzillet et al. 2021). In contrast, one study showed that a higher abundance of *F. nucleatum* predicted recurrence and shorter disease-free survival in patients with laryngeal cancer (Hsueh et al. 2022). This disagreement between studies may, however, result from variations in the tumor microenvironment between the larynx and oral cavity. Nevertheless, the capacity of *F. nucleatum* EVs to induce cancer cell migration is consistent with previous studies. *F. nucleatum* promoted OSCC cell migration by downregulating p53 and E-cadherin (Kamarajan et al. 2020) and *nucleatum*-derived EVs promoted migration and invasion of OSCC in vitro and metastasis in mice (Chen et al. 2023). Also, another study found that *F. nucleatum* promoted OSCC cell migration and additionally they described a change in the cell morphology of OSCC cells after a 48-hour treatment with *F. nucleatum* (Da et al. 2021). Changes in OSCC cell morphology by bacterial EVs was not covered by this study but in the future, it would be of importance to know if such changes can be caused by EVs too.

One important pro-tumorigenic effect of *P. gingivalis* is the ability to inhibit apoptosis in oral epithelial cells (Lee et al. 2018; Mao et al. 2007). We observed a consistent, though non-statistically significant, trend towards lower apoptosis rates in cancer cells treated with EVs from *P. gingivalis*. In addition, *P. gingivalis* has been shown to induce OSCC cell proliferation (Binder Gallimidi et al. 2015; Chang et al. 2019; Hoppe et al. 2016; Kamarajan et al. 2020), although such effect was not observed in this study. Cancer cell migration and invasion were promoted by *P. gingivalis* (Abdulkareem et al. 2018; Cho et al. 2018; Ha et al. 2016; Inaba et al. 2014; Kamarajan et al. 2020). To date, only one study has explored the effect of *P. gingivalis*-derived EVs on oral cancer, which markedly induced metastatic HSC-3 cell invasion and migration in vitro (Liu et al. 2021). We reported similar results regarding HSC-3 cell migration, but we did not observe such significant effect on cell invasion.

The oral pathogen *P. micra* has been linked to gastric (Coker et al. 2018) and colorectal cancers (Löwenmark et al. 2020; Zhao et al. 2021). Our findings support a stimulatory effect of *P. micra* EVs on the migration of metastatic cancer cells. In this regard, *P. micra* was enriched in OSCC tumor lesions, wherein the amount of *P. micra* in oral rinse sample was significantly increased from OSCC stage 1 through stage 4 patients. Thus, *P. micra* and *Parvimonas* spp. were suggested as possible parameters in biomarker panels in oral cancer (Yang et al. 2018) and for differentiating patients with oral potentially malignant disorders such as dysplasia from OSCC (W.-H. Lee et al. 2017b).

Overall, OSCC lines responded differently to the bacterial EVs. Although interesting, this is not surprising given the genetic and phenotypic differences between primary and metastatic cancer cells, including their stemness and plasticity (Salem and Salo 2023). For instance, the metastatic cancer cells exhibit high potential to acquire transitional phenotypic states, mediate drug resistance, and initiate endothelial-like capillaries (Hujanen et al. 2021; Salem and Salo 2023). However, studies on the bacterial EVs and cancer are limited, which reflects the newness of this interesting field. Therefore, the exact reason behind this behavior remains unclear and warrants further investigation. Nevertheless, it is noteworthy that bacterial species and stimulation time may play a significant role. For example, colon epithelial cells showed signs of malignant transformation when treated with CDT for 30 weeks (Guidi et al. 2013). In turn, the inducive effect of *P. gingivalis* on OSCC invasion has been reported after 30 h only (Cho et al. 2018). In two other studies, *P. gingivalis* induced a pro-malignant phenotype (i.e. EMT) in oral epithelial cells after 120 h (J. Lee et al. 2017b) and promoted primary OSCC cell invasion after 8 days (Abdulkareem et al. 2018).

We acknowledge some limitations of our study including the lack of in-depth mechanistic insight with regards to the molecular basis of EV-cancer cell interactions. Although this work lacked an in vivo model, using human-derived extracellular matrix provided some physiological relevance. In addition, challenging cancer cells with EVs, instead of bacteria, was deemed reasonable since they provide more controllable and predictable conditions compared to actual bacteria. Our EV samples had variable protein concentrations, partly due to the inherent differences across bacterial strains and their capacity to produce EVs and the variation in number of bacterial cells used during EV isolation. The particle size of the studied EVs showed modest variation (132.96–161.16 nm). It is unlikely that the variation would have affected our results since influence on particle uptake is associated with greater particle size differences, and generally spherical particles such as EVs are permissive for uptake by host cells (Baranov et al. 2020). Also, the effect of EV dose- and time-dependency and LPS detoxification warrant more studies in the future. Utilizing EVs as therapeutic agents represents a promising and rapidly emerging area in cancer research (Li et al. 2020).

## Conclusion

The present comparative study demonstrates that EVs from periodontopathogens might have the potential to influence the behavior of oral cancer cells with either inhibitory or stimulatory effects. In particular, our in vitro findings reveal a possible antitumorigenic effect of *A. actinomycetemcomitans*-derived EVs on metastatic OSCC, which encourage further in vivo studies.

### Electronic supplementary material

Below is the link to the electronic supplementary material.

Supplementary Material 1

## Data Availability

Data is provided within the manuscript or supplementary information files and available from the lead authors upon reasonable request.
