# Supplementary material for: Extracellular vesicles from Aggregatibacter actinomycetemcomitans exhibit potential antitumorigenic effects in oral cancer: a comparative in vitro study
Source: Arch Microbiol. 2024 May 3;206(6):244. doi: 10.1007/s00203-024-03976-8 (PMC11068833; doi:10.1007/s00203-024-03976-8)
Supplement: Supplementary file 1 — Supplementary Material 1 [file 203_2024_3976_MOESM1_ESM.pdf]

Extracellular vesicles from *Aggregatibacter actinomycetemcomitans* exhibit potential antitumorigenic effects in oral cancer: A comparative in vitro study

Marjut Metsäniitty[1], Shrabon Hasnat[1], Carina Öhman[2], Tuula Salo[1], Kari K. Eklund,[3,4] Jan Oscarsson[2], Abdelhakim Salem[1,3,#]

[1]Department of Oral and Maxillofacial Diseases, Clinicum, University of Helsinki, 00014 Helsinki, Finland
[2]Oral Microbiology, Department of Odontology, Umeå University, 90187 Umeå, Sweden
[3]Department of Rheumatology, University of Helsinki and Helsinki University Hospital, Helsinki, 00014, Finland
[4]Translational Immunology Research Program (TRIMM), Research Program Unit (RPU), University of Helsinki, 00014 Helsinki, Finland
[#]Corresponding author: Assoc. Professor Abdelhakim Salem (E-mail: abdelhakim.salem@helsinki.fi),
ORCID: 0000-0002-9455-3823

Supplementary figures

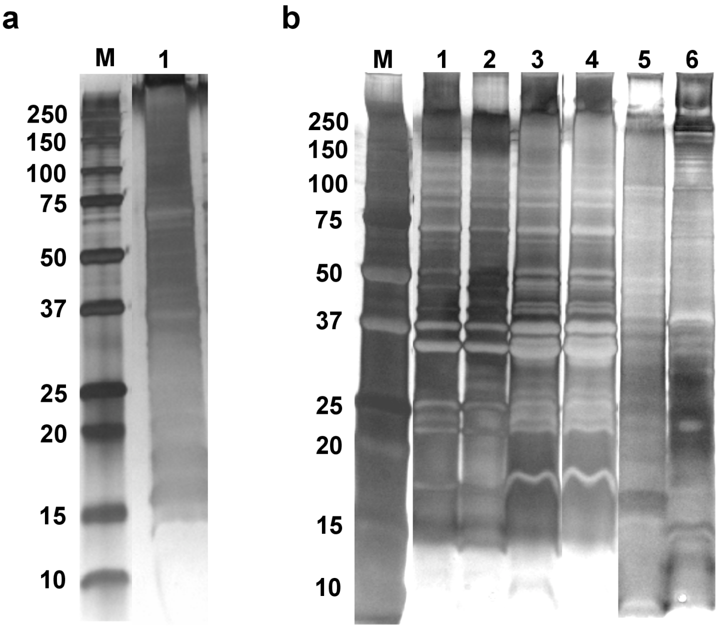

Fig. S1 SDS-PAGE analysis of EV preparations using silver staining. On the left side are protein sizes (kD) in the pre-stained molecular weight marker (M). **(a)** *P. micra* (1). **(b)** *A. actinomycetemcomitans* D7SS-WT (1). *A. actinomycetemcomitans* D7SS-*cdt* (2). *A. actinomycetemcomitans* SA3138-WT (3). *A. actinomycetemcomitans* SA3139-LPS-O (4). *P. gingivalis* (5). *F. nucleatum* (6)

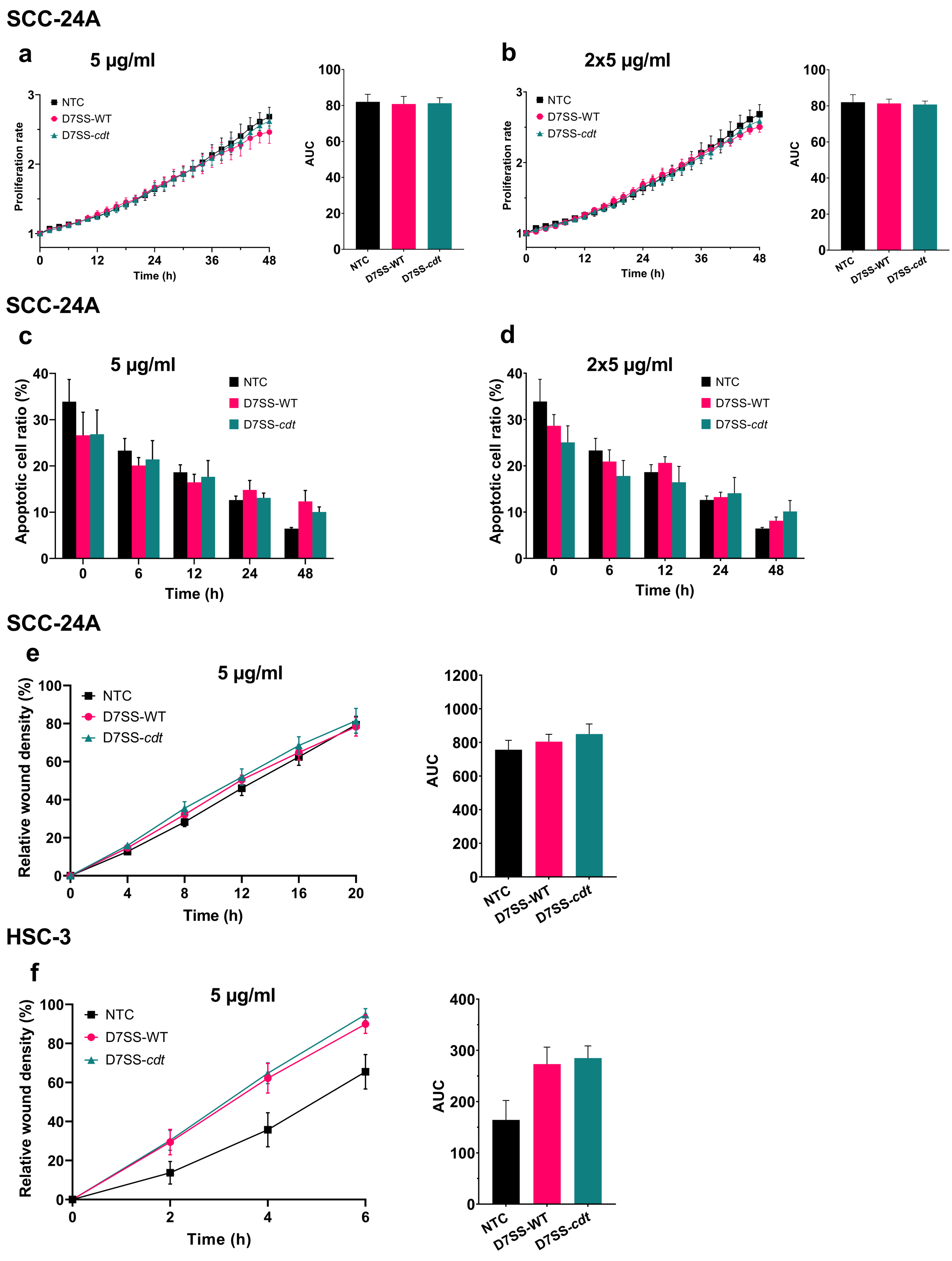

Fig. S2 Effect of *A. actinomycetemcomitans* D7SS-WT and D7SS-*cdt*-derived EVs on cancer cell proliferation, apoptosis, and migration. **(a-d)** Cell proliferation rates are presented as proliferation rate in relation to time with the corresponding area under the curve (AUC).

Apoptotic cell ratios are shown from representative time points. **(a, b)** EVs (5 µg/ml or 2×5 µg/ml) did not affect the proliferation of SCC-24A cells. **(c, d)** After 48 hours of treatment, EVs might slightly increase the number of apoptotic cells, not statistically significant difference. **(e, f)** Cell migration is presented as the relative wound density over time with the corresponding area under the curve (AUC). **(e)** EVs (5 µg/ml) did not affect the SCC-24A cell proliferation. **(f)** EVs (5 µg/ml) might increase the migration of HSC-3 cells but the difference was not statistically significant. Values are shown as mean ± SEM. NTC, no treatment control. Experiments were repeated independently three times with triplicates for each condition

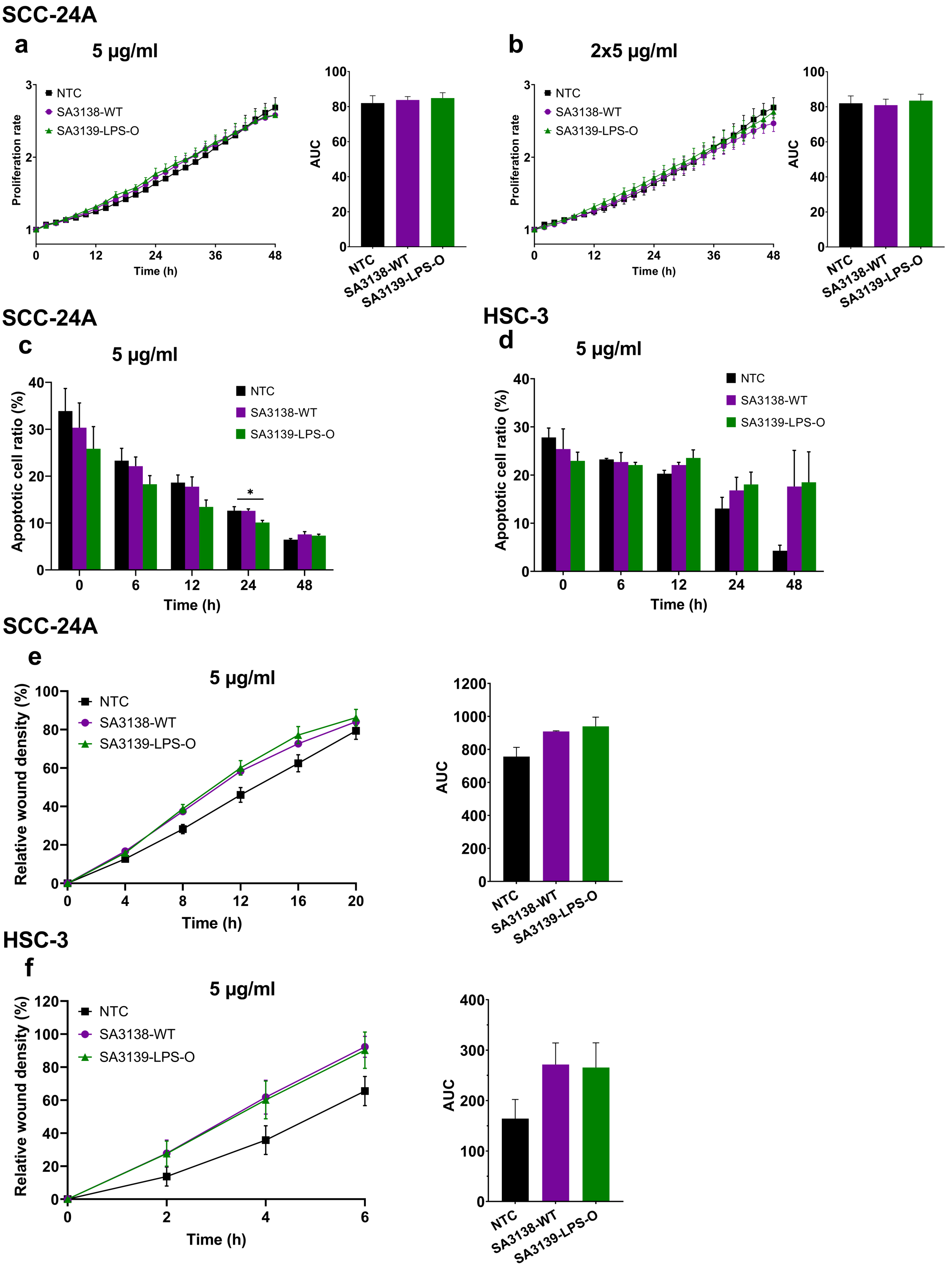

Fig. S3 Effect of *A. actinomycetemcomitans* SA3138-WT and SA3139-LPS-O-derived EVs on cancer cell proliferation, apoptosis, and migration. **(a-d)** Cell proliferation rates are presented as proliferation rate in relation to time with the corresponding area under the curve (AUC). Apoptotic cell ratios are shown from representative time points. **(a, b)** EVs (5 µg/ml or 2×5

μg/ml) did not affect the proliferation of SCC-24A cells. **(c)** The number of apoptotic SCC-24A cells was lower at 24 hours following the treatment with SA3139-LPS-O EVs (5μg/ml) compared to control cells. **(d)** The metastatic HSC-3 cell apoptosis was increased in cells treated with SA3138 and SA3139-LPS-O EVs (5μg/ml) but the difference was not statistically significant. **(e, f)** Cell migration is presented as the relative wound density over time with the corresponding area under the curve (AUC). SCC-24A and HSC-3 cells treated with EVs (5μg/ml) derived from SA3138 and SA3139-LPS-O did not show statistically significant increase in cell migration. Values are shown as mean ± SEM. $*P \leq 0.05$. NTC, no treatment control. Experiments were repeated independently three times with triplicates for each condition.

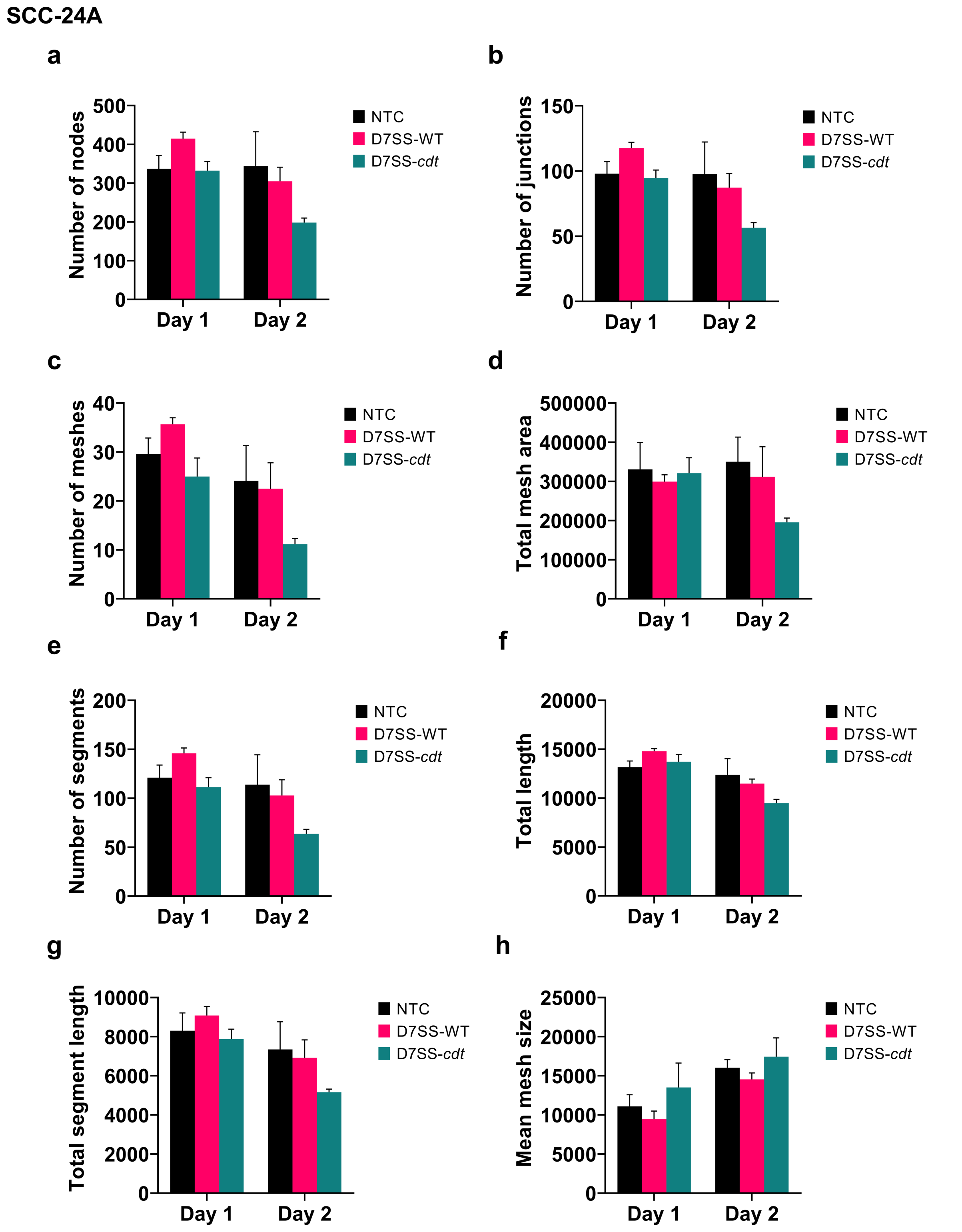

Fig. S4 Cancer cell-derived tubulogenesis in SCC-24A treated with EVs from *A. actinomycetemcomitans* D7SS-WT and D7SS-*cdt*. Images were taken on day 1 (8 h) and day 2 (20h). **(a)** Number of nodes. **(b)** Number of junctions. **(c)** Number of meshes. **(d)** Total mesh

area. **(e)** Number of segments. **(f)** Total length. **(g)** Total segment length. **(h)** Mean mesh size. Values are shown as mean ± SEM. NTC, no treatment control. Experiments were repeated independently three times with duplicates for each condition.

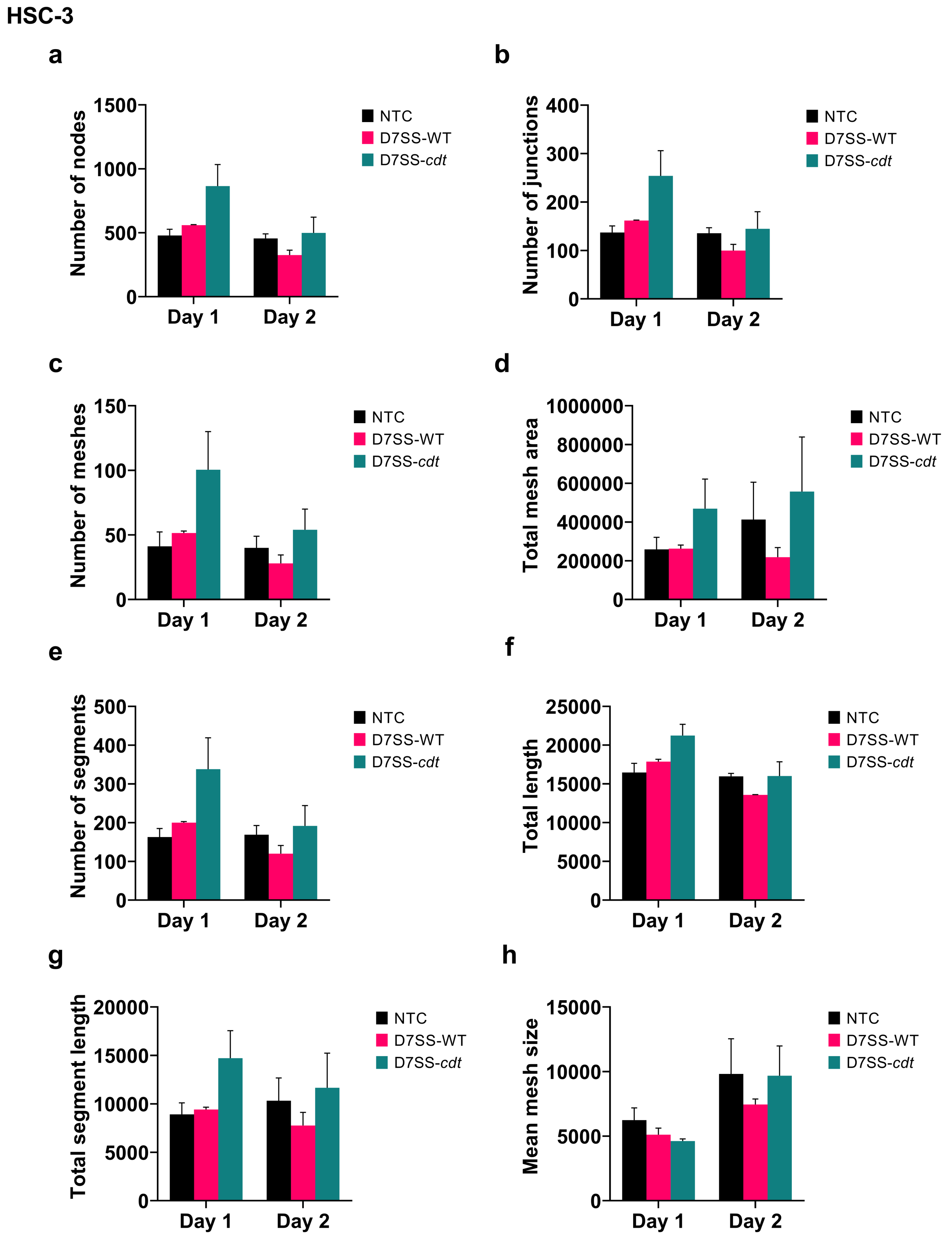

Fig. S5 Cancer cell-derived tubulogenesis in HSC-3 cells treated with EVs from *A. actinomycetemcomitans* D7SS-WT and D7SS-*cdt*. Images were taken on day 1 (8 h) and day 2 (20h). **(a)** Number of nodes. **(b)** Number of junctions. **(c)** Number of meshes. **(d)** Total mesh area. **(e)** Number of segments. **(f)** Total length. **(g)** Total segment length. **(h)** Mean mesh size.

Values are shown as mean ± SEM. NTC, no treatment control. Experiments were repeated independently three times with duplicates for each condition.

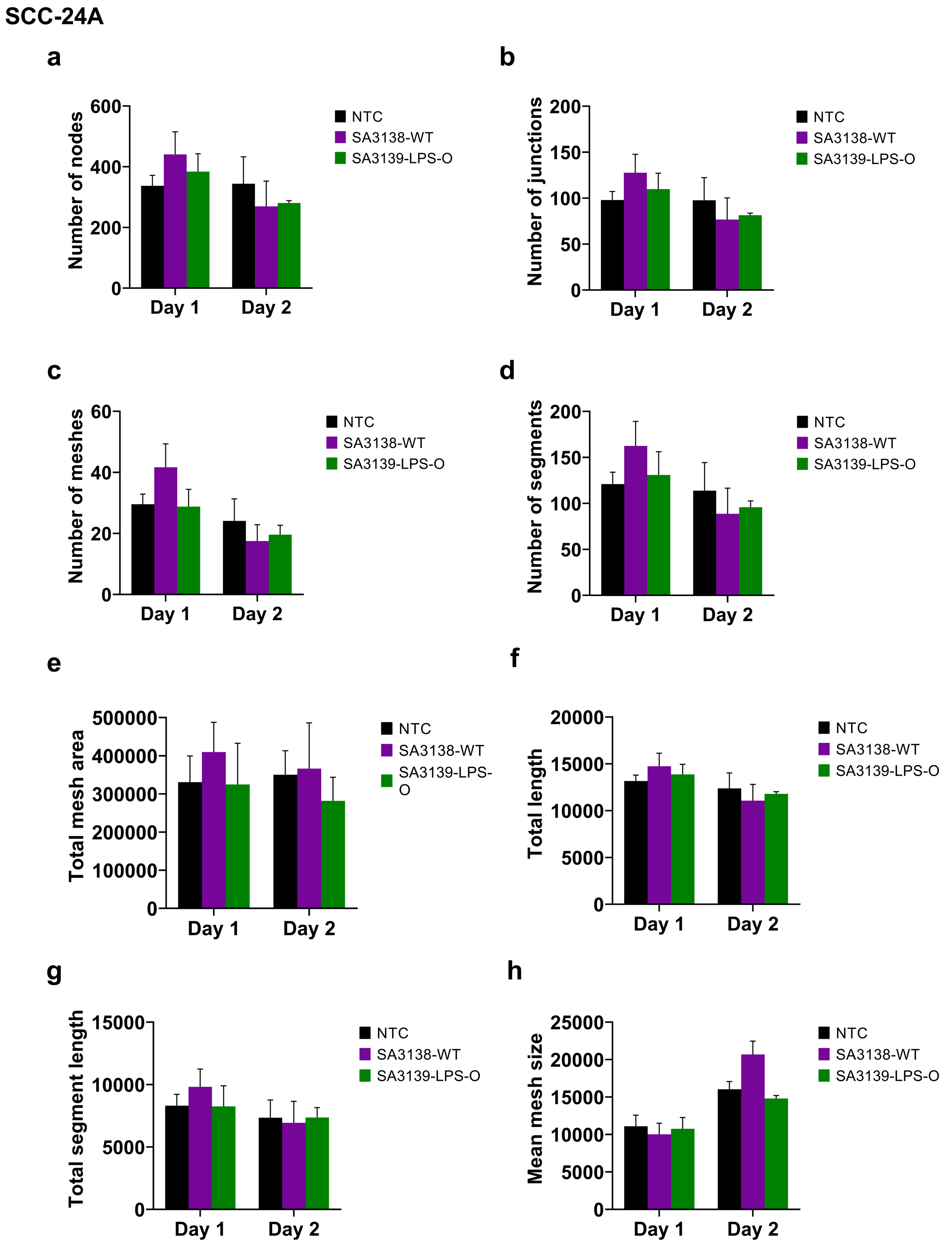

Fig. S6 Cancer cell-derived tubulogenesis in SCC-24A treated with EVs from *A. actinomycetemcomitans* SA3138-WT and SA3139-LPS-O. Images were taken on day 1 (8 h) and day 2 (20h). **(a)** Number of nodes. **(b)** Number of junctions. **(c)** Number of meshes. **(d)**

Total mesh area. **(e)** Number of segments. **(f)** Total length. **(g)** Total segment length. **(h)** Mean mesh size. Values are shown as mean ± SEM. NTC, no treatment control. Experiments were repeated independently three times with duplicates for each condition.

HSC-3

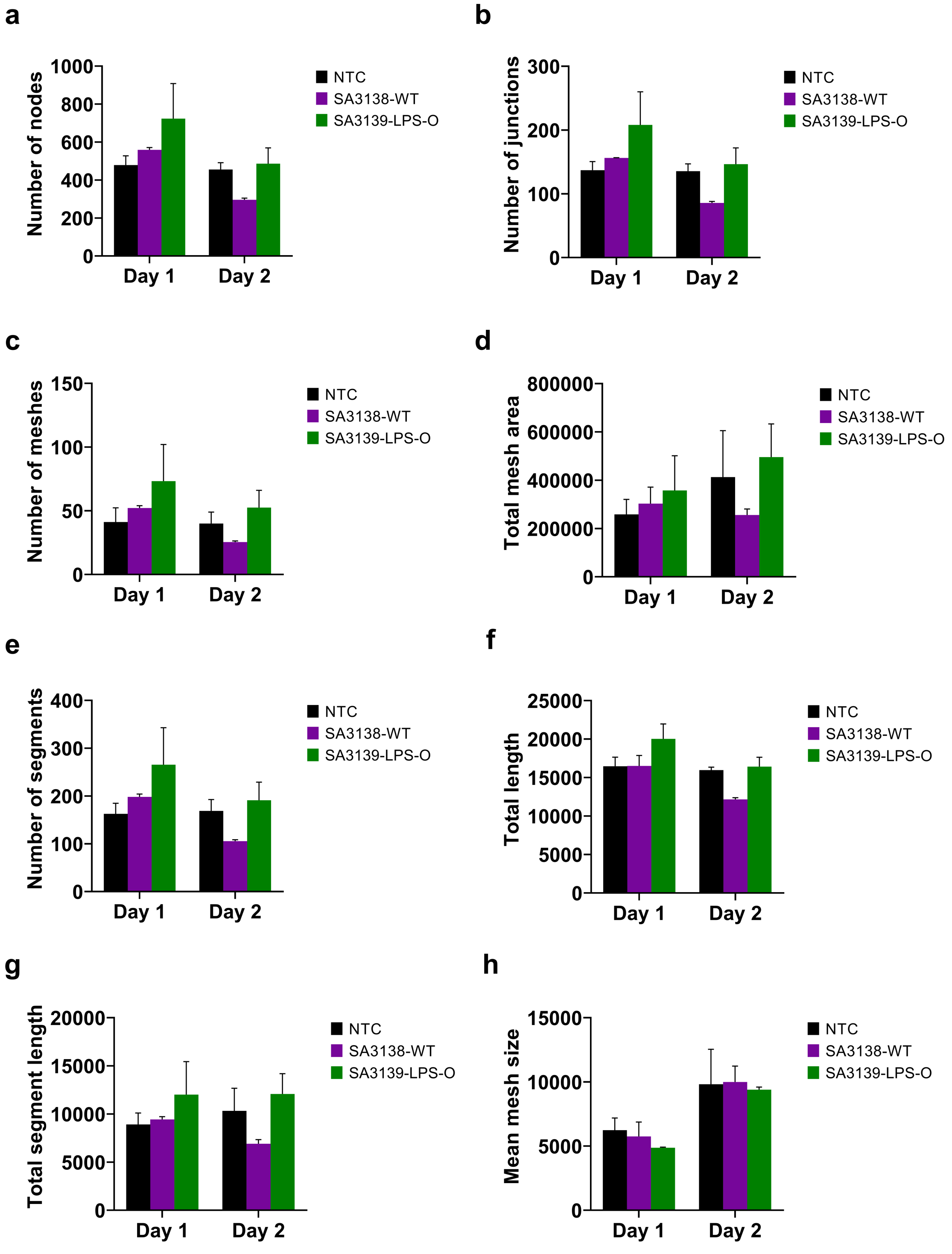

Fig. S7 Cancer cell-derived tubulogenesis in HSC-3 cells treated with EVs from *A. actinomycetemcomitans* SA3138-WT and SA3139-LPS-O. Images were taken on day 1 (8 h) and day 2 (20h). **(a)** Number of nodes. **(b)** Number of junctions. **(c)** Number of meshes. **(d)** Total mesh area. **(e)** Number of segments. **(f)** Total length. **(g)** Total segment length. **(h)** Mean

mesh size. Values are shown as mean ± SEM. NTC, no treatment control. Experiments were repeated independently three times with duplicates for each condition.

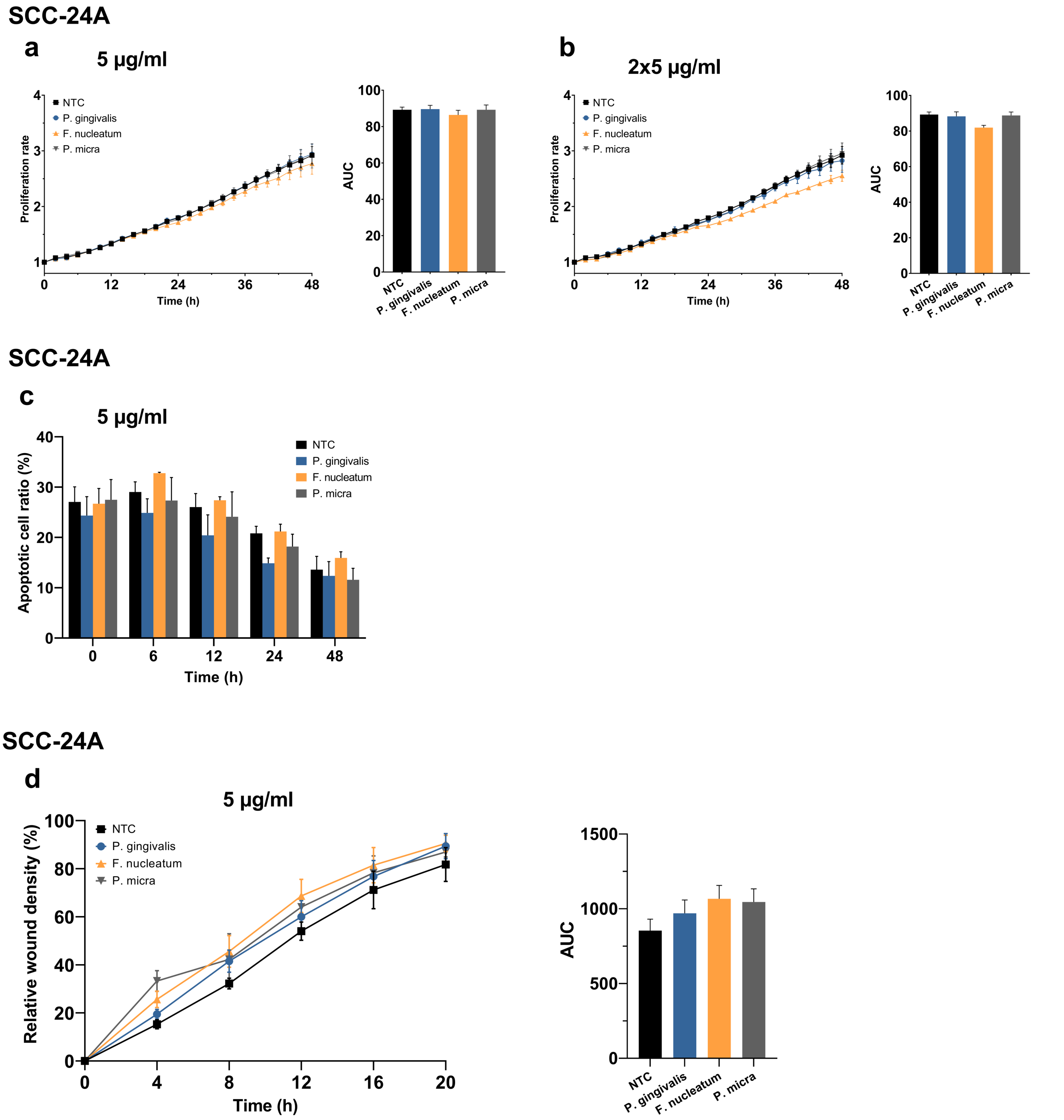

Fig. S8 Effect of *P. gingivalis, F. nucleatum* and *P. micra* -derived EVs on cancer cell proliferation, apoptosis, and migration. **(a-c)** Cell proliferation rates are presented as proliferation rate in relation to time with the corresponding area under the curve (AUC). Apoptotic cell ratios are shown from representative time points. **(a)** EVs (5µg/ml) derived from *P. gingivalis*, *F. nucleatum* or *P. micra* did not influence SCC-24A cell proliferation or apoptosis. **(b)** F. nucleatum -derived EVs (2×5 µg/ml) might decrease SCC-24A cell proliferation but the difference was not statistically significant compared to control cells (P =

0.058). **(c)** EVs (5µg/ml) derived from *P. gingivalis, F. nucleatum* or *P. micra* did not influence SCC-24A cell apoptosis. **(d)** Cell migration is presented as the relative wound density over time with the corresponding area under the curve (AUC). EVs (5µg/ml) derived from *P. gingivalis, F. nucleatum* or *P. micra* did not influence SCC-24A cell migration. Values are shown as mean ± SEM. NTC, no treatment control. Experiments were repeated independently three times with triplicates for each condition.
